# Magnitude and Associated Risk Factors of Superficial Skin Fungal Infection Among Primary School Children in Southern Tanzania

**DOI:** 10.7759/cureus.2993

**Published:** 2018-07-18

**Authors:** Rahel Chikoi, Helmut A Nyawale, Fabian P Mghanga

**Affiliations:** 1 Department of Community Medicine, Archbishop James University College, Songea, TZA; 2 Department of Microbiology and Immunology, Archbishop James University College, Songea, TZA; 3 Department of Internal Medicine, Archbishop James University College, Songea, TZA

**Keywords:** skin fungal infection, prevalence, school-children

## Abstract

Introduction: Superficial skin fungal infections are among the neglected communicable diseases in many developing countries. Schoolchildren are among the most affected groups in Southern Tanzania. The main objective of this study was to determine the magnitude and associated risk factors of superficial skin fungal infections among primary schoolchildren in Southern Tanzania.

Methods: A cross-sectional descriptive community-based study was conducted in October 2017 in a public primary school in Songea Municipal, Southern Tanzania. A sample of 500 pupils was interviewed and a physical examination performed to assess for the presence of clinically suspected skin fungal infections. Data were analyzed by SPSS v21 (IBM Corp., Armonk, NY, US).

Results: A total of 500 pupils (52.40% males) with a mean age of 9.92±1.13 years were recruited. The point-prevalence of skin fungal infections was 35.20%. Tinea capitis was the leading suspected skin fungal disease found in 73 (80.22%) pupils. Age between 10 and 12 years and sharing of a bed with more than three people were significantly associated with the development of superficial skin fungal infections (p<0.05).

Conclusion: Our findings indicate that the magnitude of superficial skin fungal infections among schoolgoing children in this study area is moderately high. We recommend the provision of health education programs for preventing and controlling diseases in schoolchildren, with the aim to reduce long-term morbidity and the socioeconomic impact.

## Introduction

Superficial skin fungal infections are fungal diseases that involve the skin, nails, mucous membrane, and hair [1]. The causative fungi have the ability to invade the superficial layers of the skin and the high keratin-concentration containing appendages, the hair and nails of the living host [2].

The condition is very common in schoolchildren and causes morbidity and lowers the quality of life of the affected children [3-4], posing a major public health problem causing, among others, poor attendance among school children in low- and middle-income countries like Tanzania. Factors such as poor personal hygiene, frequent human contact, poor environmental sanitation, overcrowding, and low socioeconomic status predispose school-age children to fungal infections [1,5-6].

Reports show that approximately 20%-25% of people worldwide are suffering from the diseases [7]. Studies from East and Southern parts of Africa shows that the prevalence of superficial skin fungal infections among schoolchildren in developing countries ranges between 20% and 90% [8]. In Tanzania, previous studies have documented prevalence rates of skin fungal infections among school-age children ranging from 12% to 55% [9-12]. The differences in prevalence are greatly attributed to differences in climatic and other geographical conditions in the studied areas.

The present study area also differs from previously studied regions in terms of climatic conditions; thus, it is imperative to assess the magnitude of skin fungal infections in this setting. This study was, therefore, designed to determine the current point-prevalence and associated risk factors of superficial skin fungal infections among primary school children in Southern Tanzania.

## Materials and methods

Study design and setting

This was a descriptive cross-sectional prospective study conducted in October 2017 in a public primary school in Songea municipal council.

Sampling technique

A list of all eight public and private primary schools was obtained from the municipal education authority. A multistage sampling technique was used that involved selection along primary schools, classes, and pupils from selected class strata. Only one school was selected from which a total of 500 out of 589 pupils from class 1 to class 7 were recruited regardless of their ages.

Data collection and clinical assessment

Selected children were interviewed and examined in daylight. All recruited pupils were interviewed to obtain their sociodemographic profiles. History to identify predisposing risk factors was also obtained. The socioeconomic status of their parents/guardians was determined using the modified wealth index [13]. The Canadian crowding index was used to determine the household living conditions of children [14]. To determine the presence of any superficial skin fungal infection, all participating pupils were examined thoroughly from head to toe with minimal clothing in a well-lit office within the school premises, and any observed fungal infection was then classified. To ensure privacy, the examinations were done in a separate private office in front of either one of their teachers of the same sex or the children's parents/guardians. The World Health Organization (WHO) hand and skin hygiene assessment tool was also used to assess the children's level of hygiene. A diagnosis of superficial skin fungal infection was mainly made clinically, and skin scrapings or nail clippings were taken to confirm the diagnosis. Pupils who had superficial skin fungal infections were treated accordingly by the examining doctors and preventive education about risk factors was provided to both the children and their parents/guardian.

Ethical issues

Ethical clearance was obtained from the Institutional Research Ethics Committee, and permission to collect data was sought from the Municipal Executive Director, municipal education authority, and the school authority. The purposes and benefits of the study were explained to the pupils, parents/guardians, and teachers. Informed written consent from the parents/guardians of all pupils involved in the study was obtained. We excluded all pupils whose parents did not give consent and those who did not assent to participate.

Statistical analysis

Statistical analyses were done using Statistical Package for Social Sciences version 21 (SPSS Inc., Chicago, IL, USA). Continuous data were expressed as means ± standard deviation (SD) and categorical data as percentages. The χ2 test and logistic regression analysis were used to determine associations for categorical variables. A p-value of less than 0.05 was considered statistically significant.

## Results

Socio-demographic characteristics and point prevalence of skin fungal infection

A total of 500 out of the 589 pupils registered in the school were recruited for the study, with a response rate of 100%. The age range was seven to 15 years and the mean age of all pupils was 9.92±1.13 years. Males were 262 (52.40%) and female pupils were 238 (47.60%). The majority of children belonged to the age group of 10 to 12 years (298, 59.60%). Class 4 pupils formed the majority of the participants (140, 28.00%) while about 62.60% of the pupils' parents/guardian were married. Out of the 500 pupils, 176 pupils were found to have a superficial fungal infection giving a point-prevalence of 35.20%. Females (59.09%) were more affected than males (40.91%), and this difference was statistically significant (**p <0.05**). The highest prevalence was found among the age group 10–12 years (**p<0.05**) and among pupils of classes 3 and 4 (**p<0.05**) (Table 1).

**Table 1:** Socio-demographic characteristics of study subjects

Variable	Total number, N (%)	Status of Skin Fungal Infections		p-value
		Present, n (%)	Absent, n (%)	
Sex				
Male	262 (52.40)	72 (40.91)	190 (58.64)	0.00
Female	238 (47.60)	104 (59.09)	134 (41.36)	
Age (years)				
7 – 9	128 (25.60)	49 (27.84)	79 (24.38)	
10 – 12	298 (59.60)	92 (52.27)	206 (63.58)	0.03
13 – 14	70 (14.00)	34 (19.32)	36 (11.11)	
15+ and above	4 (0.80)	1 (0.57)	3 (0.93)	
Class				
One & Two	143 (28.60)	44 (25.00)	99 (30.56)	
Three & Four	163 (32.60)	94 (53.41)	69 (21.30)	0.00
Five & Six	146 (29.20)	28 (15.91)	118 (36.42)	
Seven	48 (9.60)	10(5.68)	38 (11.72)	
Parents' marital status				
Married	313 (62.60)	108 (61.36)	205 (63.27)	
Single	72 (14.40)	25 (14.21)	47 (14.51)	
Cohabiting	24 (4.80)	9 (5.11)	15 (4.63)	0.71
Polygamy	48 (9.60)	21 (11.93)	27 (8.33)	
Divorced	43 (8.60)	13 (7.39)	30 (9.26)	
Parents' occupations				
Peasant	118 (23.60)	73 (41.48)	45 (13.89)	
Employed	190 (38.00)	51 (28.98)	139 (42.90)	0.00
Businessmen/women	172 (34.40)	47 (26.70)	125 (38.58)	
Others	20 (4.00)	5 (2.84)	15 (4.63)	
Socio-economic status (SES)				
Low SES	433 (86.60)	141 (80.11)	292 (90.12)	
Middle SES	39 (7.80)	23 (13.07)	16 (4.94)	0.00
High SES	5 (1.00)	1 (0.57)	4 (1.24)	
Unknown	23 (4.60)	11 (6.25)	12 (3.70)	

Clinical types of dermatophytosis lesions

**Figure 1:**
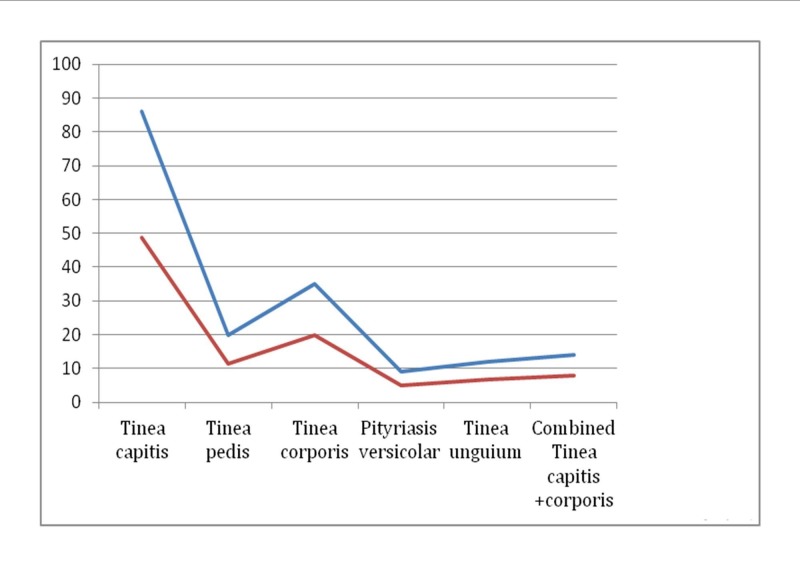
Clinical types of skin fungal infection lesions among study participants The red line represents the proportions of children with clinical types of dermatophytosis and the blue line shows the actual number of children with clinical types of dermatophytosis.

Of the 500 pupils who underwent the physical examination for skin fungal infections, 176 (35.20%) were identified to have one or more types of suspected skin fungal infections lesions. Of these, Tinea capitis was the leading suspected skin fungal infection found in 86 children (48.86%), followed by Tinea corporis, which was found in 35 pupils (19.89%). Multiple infections of two types of dermatophytosis lesions were observed in 14/176 pupils (7.95%) (Figure 1). 

Assessment of personal body hygiene

We assessed the habits and behaviors of pupils regarding care of their skins. Of the 500 children interviewed, 376 (75.20%) use safe tap-water for washing their clothes and bathing. About 440 (88%) of the children wash their bodies only once a week. Furthermore, about 115 (23%) pupils do not use soap when taking a bath. Almost 475 (95%) pupils share combs and about 453 (90.60%) share towels with their siblings or relatives (Table 2).

**Table 2:** Personal hygiene and skin care-related habits among study subjects

Risk factors	Number, N	Percentage (%)
Source of water		
Tap water	376	75.20
Well water	83	16.60
Other sources	41	8.20
Frequency of taking showers		
Daily	17	3.40
More than twice a week	43	8.60
Once a week	440	88.00
Using soap during showers		
Yes	334	66.8
Occasionally	51	10.20
Never	115	23.00
Number of people per room		
1 – 3	417	83.40
4 – 10	61	12.20
>10	22	4.40
Number of people sharing a bed with the child		
Sleeping alone	128	25.60
2	300	60.00
>3	72	14.40
Comb sharing		
Never	11	2.20
Yes	475	95.00
Occasionally	14	2.80
Towel sharing		
Never	5	1.00
Yes	453	90.60
Occasionally	42	8.40
Sharing of clothes and shoes		
Never	39	7.80
Yes	446	89.20
Occasionally	15	3.00

Risk factors for skin fungal infections among study subjects

We also performed a multivariate logistic regression analysis to determine the association between variables and the presence of fungal infection among children. The variables age between 10 and 12 years (p<0.05) and between 13 and 14 years (p<0.001), class of pupils (p<0.05), having parents who are employed or dealing with business (p<0.001), having parents of middle socioeconomic status (p<0.001), having a shower using water from other sources (p<0.001), not taking a shower daily (p<0.05), not using soap or using it occasionally when taking a shower (p<0.001), staying or sleeping with more than three people in the same room (p<0.001), and sharing a bed with more than three people, were all significantly associated with the development of superficial skin fungal infections (Table 3).

**Table 3:** The logistic regression analysis of the risk factors for acquiring superficial skin fungal infections among pupils (n=500) Abbreviations: n, positive number; OR, odds ratio; CI, confidence interval

Predictor variables	Status of skin fungal infection			
	Present, n	Absent, n	OR (95% CI)	p-value
Age (years)				
7 – 9	49	79	1.00	
10 – 12	92	206	0.63 (043 – 0.91)	0.02
13 – 14	34	36	2.12 (1.27 – 3.52)	0.00
15+ and above	1	3	0.61 (0.06 – 5.92)	1.00
Class				
One & Two	44	99	1.00	
Three & Four	94	69	4.24 (2.85 – 6.31)	0.00
Five & Six	28	118	0.33 (0.21 – 0.52)	0.00
Seven	10	38	0.45 (0.22 – 0.93)	0.04
Parents' marital status				
Married	108	205	1.00	
Single	25	47	0.98 (0.58 – 1.65)	1.00
Cohabiting	9	15	1.11 (0.48 – 2.59)	0.83
Polygamy	21	27	1.49 (0.82 – 2.72)	0.21
Divorced	13	30	0.78 (0.40 – 1.54)	0.51
Parents' occupations				
Peasant	73	45	1.00	
Employed	51	139	0.54 (0.37 – 0.80)	0.00
Businessmen/women	47	125	0.58 (0.39 – 0.87)	0.00
Others	5	15	0.60 (0.22 – 1.69)	0.36
Socio-economic status (SES)				
Low SES	141	292	1.00	
Middle SES	23	16	2.89 (1.49 – 5.64)	0.00
High SES	1	4	0.46 (0.05 – 4.12)	0.66
Unknown	11	12	1.73 (0.75 – 4.01)	0.26
Source of water				
Tap water	100	276	1.00	
Well water	51	32	3.72 (2.28 – 6.07)	8.16
Other sources	25	16	3.19 (1.65 – 6.18)	0.00
Frequency of taking showers				
Daily	1	16	1.00	
More than twice a week	11	43	0.44 (0.22 – 0.87)	0.02
Once a week	164	276	2.38 (1.23 – 4.61)	0.00
Using soap during showers				
Yes	28	306	1.00	
Occasionally	47	4	29.15 (10.29 – 82.56)	0.00
Never	101	14	29.82 (16.15 – 55.06)	0.00
Number of people per room				
1 – 3	108	309	1.00	
4 – 10	49	12	10.03 (5.16 – 19.49)	0.00
>10	19	3	12.95 (3.78 – 44.41)	0.00
Number of people sharing a bed with the child				
Sleeping alone	12	116	1.00	
2	96	204	0.71 (0.49 – 1.02)	0.07
>3	68	4	50.37 (17.95 – 141.34)	0.00
Comb sharing				
Never	1	10	1.00	
Yes	170	305	1.77 (0.69 – 4.50)	0.29
Occasionally	5	9	1.02 (0.34 – 3.10)	1.00
Towel sharing				
Never	1	4	1.00	
Yes	156	297	0.71 (0.39 – 1.30)	0.34
Occasionally	19	23	1.58 (0.84 – 3.00)	0.18
Sharing of clothes and shoes				
Never	11	28	1.00	
Yes	156	290	0.91 (0.51 – 1.64)	0.88
Occasionally	9	6	2.86 (1.00 – 8.16)	0.05

## Discussion

This community-based cross-sectional study described the magnitude and risk factors of skin fungal infections among primary schoolchildren at a public school in Southern Tanzania. The study recruited 500 pupils with an almost equal male: female enrollment (1:1.1). The pupils' mean age was 9.92±1.13 years with the majority of the pupils belonged to the age group 10–12 years. The studied age group is the most affected population worldwide, and this profile is in line with those reported elsewhere [10-12,15] and is within the reference school-age range of six to 12 years. Significantly more girls were found to have skin fungal infections than boys. This is a surprising finding because boys are more hyperactive than girls, thus one would expect them to be more infected than girls. The possible explanation for this contra-observation could be related to the culture of the local people who involve young girls in participating in household work, which could likely predispose them to skin infections. We also observed that the infections affected more children from families with a low social economic status and peasants parents. This observation is in line with suggestions that superficial skin fungal infections are diseases of poverty and tend to run through poor communities.

The present study found a point-prevalence of superficial skin fungal infections of 35.20%. This prevalence lies between those previously reported by other studies done in the country [9-12] and elsewhere [5-7]. This moderately high prevalence reflects unhygienic environmental health conditions that favor the occurrence of skin fungal infections in this study population. This finding is also an indication of the poor socioeconomic status prevailing in the study area. Kalu et al. [15], Oyedeji et al.[16], and Saheed et al. [17] all reported similar environmental conditions in their study areas, which included the low socioeconomic status of communities, poor personal hygiene practices among children, low level of education, inadequate environmental health practices, poor water distribution, and adverse socio-cultural practices. As skin fungal infections are a disease of poverty, much needs to be addressed by the respective communities to curb the problem.

Five clinical types of suspected fungal infections were identified by clinical examination. Tinea capitis was the most prevalent suspected skin fungal infection observed in 49% of the pupils followed by Tinea corporis observed in 20% of the pupils. About 8% of pupils had multiple clinical types of dermatophytosis lesions. Our findings are contrary to those reported by Jena in India who reported that where pityriasis versicolor affected more than 30% of the children between infancy and age 14 years [18], while Guan-Yu Chen et al. in Taiwan reported contrary findings, where Tinea nigra and Tinea versicolor accounted for 0.09% of the clinical dermatophytosis, respectively [19]. The differences could be attributed to the geographical and climatic differences between the study areas in which the present study area has mostly long-rainy seasons and cold weather contrary to India and Taiwan, which have hot seashores and dry environments that facilitate sweating and the attachment of the organism to the skin surface. The head and body being relatively exposed parts are likely the most commonly afflicted areas by Tinea capitis and Tinea corporis in this setting, probably due to such factors as overcrowding of children in classes, frequent body contact with their friends, and poor personal body hygiene. Being mostly asymptomatic and harmless, the condition may be ignored by the pupils and their parents and, hence, can easily be further transmitted to other pupils.

We identified various factors as being significant for the occurrence of skin fungal infections in this study population. They included sociodemographic characteristics, such as age of pupils of less than 14 years, class, parents being employed or businessmen/women, and parents being in the middle economic status. Behavioral factors, such as using other sources of water for bathing and washing clothes, not taking shower daily, not using soap during showers, staying or sleeping in overcrowded rooms, and sharing a bed with more than three people. Our findings are similar to those reported elsewhere [5-7,11,15].

Contrary to our findings, Figueroa et al. in their study in South Western Ethiopia found overcrowding not to be significantly associated with infection [6]. Similar findings were also reported by Olutoyin et al. in South Western Nigeria who observed an increased prevalence of skin fungal infections in the urban community than in the rural community and that more people who live in crowded conditions had skin fungal infections than those living in less populated homes although this relationship was not statistically significant [20]. Our findings are in line with the well-known fact that skin fungal infections, being transmitted mainly through skin-to-skin contact, tend to be prevalent in areas where there are overcrowding and possible frequent skin contacts between children.

Children whose parents were employed or were dealing with business had a significantly higher burden of the disease than their counterparts. Because of their parents' occupation, it is probable that these children lacked proper and close supervision of their health and effective care, such as good personal hygiene, taking regular showers, and even better personal grooming than their age group. However, our findings are contrary to those reported by Olutoyin who reported low incidences of skin infections among children who stay with parents of similar status [20]. Nevertheless, proper and close supervision of children's behaviors and habits leading to personal body hygiene are key to the prevention of skin fungal infections.

Parents' socioeconomic status has been one of the main factors associated with the development of superficial fungal infections among pupils. Previous studies have shown that children with parents of low socioeconomic status were reported to have an increased prevalence of superficial fungal infections [6-7,10-12,17,20]. On the contrary, our study showed that a middle socioeconomic status was significantly associated with the increasing prevalence of skin fungal infections than a low socioeconomic status. Despite this finding, poverty is one of the main reasons perpetuating the existence of skin fungal infections in this study environment. Pupils from families with a high or middle socioeconomic status are likely to observe and practice hygienic principles than are pupils from poor families.

Sharing of clothes, combs, towels, or shoes have been reported elsewhere to be good sources of the transmission of superficial fungal infection [6-7,10-12,20]. In this present study, although Tinea capitis and Tinea corporis were the most observed prevalent clinical types, there was no significant association between the sharing of the items with the development of skin fungal infections. Most of the pupils are from poor families with the majority taking a bath only once a week. The sharing of such items, which are very likely to be dirty and unhygienic, poses the risk of contracting skin fungal infections among pupils.

Such factors as personal hygiene among pupils influence the transmission of superficial fungal skin infection as reported in this study and elsewhere. Our findings suggest that to prevent and control the spread of skin fungal infection, measures to improve personal hygiene among pupils should be encouraged. Strategies such as health education to both pupils and their parents/guardians can help increase awareness of the disease and change personal health behaviors among children.

Our study used only one public school with the majority of the pupils from the same area and who had more or less similar health environments, cultures, and taboos. The results, therefore, cannot be generalized to other private schools, which have children mostly from families with good or middle socioeconomic status and different cultures and health environments.

## Conclusions

Our findings indicate that the magnitude of superficial skin fungal infections among schoolgoing children is moderately high and that socio-demographic and behavioral factors profoundly influence the development and occurrence of these infections in children in this study area. Heath education programs are necessary for preventing and controlling the diseases in schoolchildren, with the aim to reduce long-term morbidity and the socioeconomic impact.
